# Dietary supplementation with hydroxy-methionine manganese improves meat quality, antioxidant capacity, and lipid metabolism in Cherry Valley ducks (*Anas platyrhynchos domesticus*)

**DOI:** 10.3389/fvets.2025.1481793

**Published:** 2025-04-04

**Authors:** Yang Liu, Qinteng Hou, Yaqi Chang, Yueqin Xie, Hua Zhao, Xiaoling Chen, Guangmang Liu, Jiayong Tang, Gang Tian, Jingyi Cai, Gang Jia

**Affiliations:** Institute of Animal Nutrition, Key Laboratory for Animal Disease-Resistance Nutrition of China, Ministry of Education, Sichuan Agricultural University, Chengdu, Sichuan, China

**Keywords:** hydroxy-methionine manganese, *Anas platyrhynchos domesticus*, lipid metabolism, antioxidant, meat quality

## Abstract

Hydroxymethionine manganese (MnHMet), as a novel organic trace element additive, has demonstrated significant effects on improving meat quality, enhancing antioxidant capacity, and lipid metabolism. However, its specific effects on Cherry Valley ducks remain unclear. This study explored the effects of dietary MnHMet on meat quality, antioxidant capacity, and lipid metabolism in meat ducks. In a 35-day study, 560 1-day-old male ducks were randomly assigned to seven groups: six groups were supplemented MnHMet at 0, 30, 60, 90, 120, and 150 mg/kg, and a group was supplemented 120 mg/kg MnSO_4_. Results showed that the 120 mg/kg MnHMet group had significantly lower triglyceride (TG) levels than the MnSO_4_ group (*P* < 0.05). Serum high-density lipoprotein cholesterol levels increased significantly in the MnHMet groups compared to the 0 mg/kg group and showed a quadratic change to increasing MnHMet levels (*P* < 0.05). MnHMet supplementation reduced drip loss, shear force, abdominal fat weight, and percentage while increasing intramuscular fat (IMF, *P* < 0.05). Drip loss and shear force decreased linearly, and IMF showed a quadratic response to MnHMet levels (*P* < 0.05). Fatty acid analysis revealed a quadratic decrease in hepatic C23:0 concentrations (*P* < 0.05). MnHMet improved antioxidant capacity by enhancing total antioxidant capacity (T-AOC), upregulating *MnSOD* mRNA expression in the liver and breast muscle, increasing hepatic MnSOD levels, and reducing malondialdehyde (MDA) levels (*P* < 0.05). T-AOC levels exhibited quadratic and linear increases in breast muscle and liver, respectively, while hepatic MDA levels decreased quadratically (*P* < 0.05). Catalase levels in breast muscle were significantly higher in the MnHMet group than in the MnSO_4_ group (*P* < 0.05). Additionally, MnHMet reduced adipocyte area, downregulated hepatic fatty acid synthase and acetyl-CoA carboxylase, and upregulated peroxisome proliferator-activated receptor-γ, carnitine palmitoyltransferase-1α, and lipoprotein lipase (*P* < 0.05). Based on IMF and abdominal fat percentage, the optimal MnHMet supplementation levels were 107.5 and 117.5 mg/kg, respectively. These results revealed that MnHMet supplementation improved muscle mass, fatty acid composition, reduced abdominal fat, and enhanced meat quality by regulating antioxidant capacity and lipid metabolism in meat ducks.

## Introduction

In China's meat consumption, duck meat holds a prominent place and is second most consumed poultry meat after chicken meat (data form National waterfowl industry technology research and Development Center). Renowned for its rich nutritional value, duck meat is favored by consumers for its abundant amino acids and polyunsaturated fatty acids, as well as its relatively low fat content (1). However, high levels of polyunsaturated fatty acids and essential amino acids also make duck meat particularly susceptible to oxidation, leading to lipid rancidity and positively correlated with protein oxidation, which may impair meat quality and flavor (2). As the demand for high-quality poultry products escalates, consumers are increasingly focusing on meat quality and flavor as well as its nutritional value by consumers (3). Feed nutrients are crucial for animal muscle quality (4). Consequently, people are increasingly interested in exploring nutritional strategies to optimize duck health and production, such as organic acids, plant extract, and trace elements (5, 6).

Manganese (Mn) is an essential trace element in animal nutrition, and poultry diets should contain at least 60 mg/kg of manganese (7). Mn can promote metabolism by activating metalloenzymes involved in the metabolism of carbohydrates, lipids, and amino acids (8). Additionally, Mn is also an important component of manganese superoxide dismutase (Mn-SOD), which can protect mitochondria from oxidative damage (9). Mn deficiency can result in reduced growth, impaired metabolism, ataxia, and compromised reproductive function, thereby ultimately impacting the overall growth and development of the body (10). In the meat production, oxidative stress is a key factor influencing muscle quality and serves as an important indicator for its evaluation (11). Recent research has shown that supplementing diets with exogenous manganese significantly improves the antioxidant capacity of poultry and pigs, thereby improving their meat quality (12–14). Therefore, additional Mn is usually supplemented in the diet to improve animal muscle quality. In the current duck production, Mn is primarily incorporated into the trace mineral premix in its inorganic form. Our recent study demonstrated that adding manganese sulfate (MnSO_4_·H_2_O) to the diet of meat ducks could improve meat quality and enhance antioxidant activity and intramuscular fat by regulating the expression of genes involved in fat synthesis and decomposition (15). However, inorganic trace elements have a high loss rate and low utilization rate, and excessive addition may cause environmental contamination due to excessive excretion (16, 17).

Research has shown that organic chelated trace minerals have higher bioavailability compared with inorganic mineral elements (18). Hydroxy-methionine manganese (MnHMet), which is a new type of organic Mn, synthesized through the chelation of hydroxy-methionine with the trace element Mn (19). Distinguished from its inorganic counterparts, MnHMet boasts a stable chemical structure with high bioavailability. Meanwhile, it not only supplements the trace elements required for animal growth, but also provides the necessary methionine. Thus, chelated Mn has been used more frequently as Mn fortification in animal nutrition. Recent research found that dietary addition of MnHMet improves the growth performance and trace element deposition in broilers (20). However, to date, there is limited literature examining the impact of dietary MnHMet supplementation in meat ducks.

This study aimed to explore the effects of dietary MnHMet supplementation on the growth performance, meat quality, antioxidant capacity, and lipid metabolism of meat ducks, and to compare and evaluate the differences between MnHMet and inorganic Mn. Our study will offer valuable insights to serve as a reference for the potential utilization of MnHMet as a novel trace element source in duck feed.

## Materials and methods

### Experimental design and management

A total of 560 one-day-old male Cherry Valley ducks with average body weight (BW) of 55.76 ± 0.27 g were purchased from a local hatchery. All ducks were selected and randomly distributed into 7 dietary treatments with 8 replicates (10 ducks per replicate/cage). The treatment groups were as follows: (1) MnHMet group, the six groups of meat ducks were supplemented with MnHMet at levels of 0, 30, 60, 90, 120, and 150 mg/kg (as Mn) in the basal diet, respectively; (2) inorganic manganese group, the group was fed a basal diet supplemented with 120 mg/kg MnSO_4_ (as Mn). The MnHMet and MnSO_4_ were provided by the Sichuan Chelota Biotech Corporation Limited (Guanghan, China). The MnSO_4_ contained 31.8% Mn, and MnHMet contained 16.8% Mn. The 35-day experiment was divided into two phases (days 1–14 and days 15–35), and the experimental basal diets (Table 1) were prepared according to the feeding standard of China for meat ducks (NY/T2122-2012). The feed provided in the form, and the diameter of pellets was 2 mm at 1 to 14 days of age and 3 mm at 15 to 35 days of age. The measured manganese content in each treatment group is shown in Supplementary Table S1. All the meat ducks were housed within three-level cages (2.0 m × 1.0 m), which were placed in a temperature and light-controlled room with constant lighting at the experimental farm. The room temperature was maintained at 33 ± 1°C for the first week and then reduced by 3°C per week until it reached 24 ± 1°C, with a relative humidity of 65%−75%. Feed and water were offered ad libitum during the experiment. On the morning of days 1, 14, and 35, all ducks were weighed by replicates to calculate the BW and average daily gain (ADG). Besides, the feed intake per replicate was recorded daily to calculate the average daily feed intake (ADFI), and the feed-to-gain ratio (F/G) was calculated.

**Table 1:** Composition and nutrient level of the basal diet (air-dry basis, %).

**Ingredients**	**Content**	
	**1–14 d**	**15–35 d**
Corn	61.06	67.07
Soybean meal	34.76	27.40
Wheat bran	0.50	2.00
Limestone	0.78	0.75
Dicalcium phosphate	2.04	1.97
Sodium chloride	0.30	0.30
Choline chloride, 50 %	0.15	0.15
Vitamin premix^a^	0.03	0.03
Mineral premix^b^	0.20	0.20
DL-Methionine	0.15	0.13
L- Lysine·HCl	0.03	0.00
Total	100.00	100.00
Nutrient level^c^		
AME, MJ/kg	12.14	12.14
Crude protein	20.02	17.51
Calcium	0.90	0.85
Available phosphorus	0.42	0.40
Lysine	1.10	0.91
Methionine	0.46	0.41
Tryptophan	0.24	0.20
Threonine	0.77	0.67

a The premix contained the following per kilogram of the diet: VA 9000 IU, VD3 2,000 IU, VE 10 mg, VB1 2 mg, VB2 4.8 mg, VB3 50 mg, VB5 20 mg, VB9 1 mg, VB12 0.02 mg.

b The premix provided the following per kilogram of the diet: Cu (CuSO_4_ 5H_2_O) 4.4 mg, Fe (FeSO_4_ 7H_2_O) 44.1 mg, Zn (ZnSO_4_ 7H_2_O) 66.06 mg, Se (NaSeO_3_) 0.3 mg.

c Calculated values.

### Samples collection

After 12 h of fasting on day 35 of the experiment, one duck from each replicate was selected. The blood samples (10 mL) were collected from the wing veins into vacuum tubes without anticoagulant. The blood samples were allowed to stand at room temperature for 30 min, followed by centrifugation at 3,000 g for 15 min at 4°C to obtain the serum, which was then stored in a refrigerator at −20°C for further analysis. Subsequently, the selected ducks were euthanized. In a sterile environment, breast muscle, leg muscle, liver, and abdominal adipose tissue were collected from the same part of each duck. The abdominal adipose tissue was excised and then subjected to multiple washes with a 9% saline solution. Following this, it was preserved in a 4% paraformaldehyde solution for histological analysis. Additionally, breast muscle, leg muscle, and liver samples were also flushed multiple times with 9% saline solution. These tissue samples were then stored at −80°C for subsequent analysis.

### Measurement of slaughter performance

After 12 h of fasting on day 35 of the experiment, another duck per replicate was selected and euthanized. Subsequently, the determination of carcass trait (carcass weight, half carcass weight, eviscerated weight, breast muscle weight, thigh muscle weight, abdominal fat weigh, dressing percentage, half carcass weight rate, eviscerated carcass weight rate, breast muscle rate, thigh muscle rate, and abdominal fat percentage) was performed according to NY/T 823-2020 Poultry Production Performance Terms and Metric Statistical Methods (China Agricultural Industry Standard, 2020) (21).

### Measurement of meat quality

The pH of the breast muscle was measured using a Testo 205 pH meter (Testo AG, Lenzkirch, Germany) at 45 min and 24 h post-slaughter. The final average value was calculated based on three measurements per sample.

The lightness (L^*^), redness (a^*^), and yellowness (b^*^) of the breast muscles were measured using a chroma meter (CR-400, Konica Minolta, Japan) at 45 min post-slaughter, and the color of the breast muscles for each sample was determined in triplicate at three different points (middle, medial, and lateral).

The meat samples to be tested were trimmed into 3 cm × 2 cm × 1 cm pieces (about 5 g, W1), placed in a sealed polyethylene bag, and stored in the freezer at 4°C for 24 h. The meat samples (W2) were reweighed after wiping off the surface moisture, and the drip loss was calculated as follows: [(W1 – W2)/W1] × 100%.

The cooked meat samples were trimmed into strips of about 1.5 cm × 1.5 cm × 1 cm, ensuring that that the muscle fibers extended in the same direction. Then the shear force of the samples was determined using a shear apparatus (C-LM3B, Harbin, China), and three values were determined for each meat sample and averaged.

Approximately 50 g of breast muscle samples were freeze-dried for 72 h, crushed, and then analyzed for crude protein and fat content using AOAC methods (22). The intramuscular fat (IMF) content was calculated by dividing the weight of fat by the total weight of the sample.

### Analysis of serum biochemical indicators and hormone levels

Serum levels of non-esterified fatty acid (NEFA), triglyceride (TG), total cholesterol (TC), high-density lipoprotein cholesterol (HDL-C), and low-density lipoprotein cholesterol (LDL-C) were analyzed using commercial kits (Nanjing Jiancheng Bioengineering Institute, Nanjing, China) on a Hitachi 7020 automatic biochemical analyzer (Hitachi Medic, Tokyo, Japan) (23).

### Observation of abdominal adipose tissue

Following a 24-h fixing period in a 4% paraformaldehyde solution, the abdominal adipose tissues were extracted and subsequently embedded using the paraffin embedding process outlined by Zhang et al. (24). The tissues were then automatically sliced into 7-μm-thick sections using the automatic slicer (HM355S, Burton International Trading Co., Ltd., China). Afterwards, Oil Red O staining was stained. For each section of the abdominal fat, three visual fields were randomly selected for image acquisition. The average diameter and area of abdominal adipocytes were measured and calculated.

### Analysis of tissue antioxidant levels

The samples of liver and breast muscle were homogenized in ice-cold saline solution (1:9, w/v), and then the supernatant was prepared after centrifugation at 2,500 r/min for 10 min at 4°C to determine the levels of liver and breast muscle antioxidant indexes. The manganese superoxide dismutase (MnSOD, hydroxylamine method), total antioxidant capacity (T-AOC, ABTS fast method), catalase (CAT, visible light method), and malondialdehyde (MDA, TBA method) contents were determined in the liver and breast muscle according to the commercial kit (Nanjing Jianjian Bioengineering Institute, Nanjing, China) instruction. For details, please refer to Du et al. and Zhao et al. (25, 26).

### Fatty acid analysis

The liver's fatty acid composition was analyzed using a GC-2010 Plus gas chromatograph (Shimadzu, Japan) equipped with an AOC-20i autoinjector (Shimadzu, Japan) and a chromatographic column SP2560 for fatty acid methyl esters (100 m × 0.25 mm id × 0.2 μm) following the previous methodology established by Xu et al. (27). Initially, ~100 mg of liver dry sample was used for lipid extraction, adopting previously detailed by Folch et al. (28). The extracted lipids underwent hydrolysis, methylation, and were dissolved in a mixture of n-hexane and saturated NaCl solution for subsequent analysis. Identification of the fatty acids was achieved by comparing peak retention times with standardized samples (Sigma, USA), and the fatty acid profile was expressed as a percentage of the total fatty acids.

### Analysis of quantitative real-time PCR (RT-qpcr)

The 50–100 mg liver and breast muscle samples were ground, and the total RNA of liver and abdominal fat samples was extracted using Trizol-reagent (Takara, Dalian, China), according to the manufacturer's instructions. Subsequently, RNA was reverse transcribed to cDNA using the PremeScript RT reagent Kit following the manufacturer's instructions (Takara, Dalian, China). Finally, the qRT-PCR was performed, according to the previous study by Liu et al. (29). All gene primer sequences are displayed in Supplementary Table S2. β*-actin* was used as an internal reference gene, and mRNA levels were calculated using the 2^−ΔΔCt^ method (6).

### Statistical analysis

SPSS 25.0 software (SPSS Inc., Chicago, IL, USA) was utilized for one-way analysis of variance (ANOVA) among the different levels of MnHMet treatment groups, and variations among the treatments were compared using Duncan's multiple range test. All data were expressed as the mean ± standard error. The statistically significant differences were considered when *P* < 0.05. Then orthogonal polynomial contrasts were employed to test the linear and quadratic effects. Additionally, 120 mg/kg MnHMet group and 120 mg/kg MnSO_4_ group were subjected to analysis by *T*-test, which were considered significant at *P* < 0.05.

## Results

### Growth performance

Table 2 showed that dietary supplementation with 120 mg/kg MnHMet significantly increased the F/G of meat ducks from 15 to 35 days compared to the 0 mg/kg MnHMet group (*P* < 0.05). Additionally, the F/G of meat ducks from 15 to 35 days showed linear and quadratic changes with increasing MnHMet levels (*P* < 0.05). Nevertheless, no significant differences were observed in BW, ADG, and ADFI, as well as in the F/G for days 1-15 and days 1-35, among the different treatments (*P* > 0.05).

**Table 2:** Effects of hydroxy-methionine manganese on growth performance of meat ducks.

**Items**	**Hydroxy-methionine manganese, mg/kg**						**MnSO_4_, mg/kg**	**SEM**	***P*** **-value**		
	**0**	**30**	**60**	**90**	**120**	**150**	**120**		**ANOVA**	**Linear**	**Quadratic**
**BW, g**											
Day 1	55.76	55.73	55.58	55.53	55.41	55.64	55.97	0.001	0.424	0.144	0.225
Day 14	623.40	614.90	602.10	609.20	625.80	621.00	630.80	0.037	0.440	0.766	0.107
Day 35	2340.00	2238.60	2190.60	2215.80	2340.50	2259.50	2305.30	0.201	0.148	0.857	0.078
**ADG, g**											
Day 1 to 14	40.55	39.94	39.04	39.55	40.67	40.35	41.10	0.003	0.441	0750	0.111
Day 15 to 35	81.93	77.33	75.48	76.50	81.70	78.05	79.73	0.009	0.165	0.762	0.094
Day 1 to 35	66.66	63.74	62.87	63.95	66.62	63.98	65.86	0.006	0.259	0.751	0.174
**ADFI, g**											
Day 1 to 14	59.24	56.01	55.84	57.19	58.82	58.51	58.86	0.005	0.285	0.554	0.062
Day 15 to 35	199.83	200.26	195.52	197.56	194.64	194.66	193.67	0.012	0.562	0.108	0.849
Day 1 to 35	143.26	144.70	140.05	141.84	140.31	140.50	139.66	0.008	0.478	0.129	0.731
**F/G, g/g**											
Day 1 to 14	1.46	1.38	1.40	1.45	1.45	1.45	1.43	0.124	0.409	0.420	0.054
Day 15 to 35	2.48^ab^	2.60^a^	2.59^a^	2.56^ab^	2.39^c^	2.41^bc^	2.44	0.023	0.013	0.025	0.029
Day 1 to 35	2.17	2.21	2.22	2.23	2.10	2.12	2.12	0.016	0.059	0.059	0.068

BW, Body weight; ADG, Average daily gain; ADFI, Average daily feed intake; F/G, Feed to gain ratio.

^a, b, c^Mean values (*n* = 6) in a row without a common superscript are significantly different in variance analysis (*P* < 0.05).

### Serum biochemical parameters

The effect of MnHMet on serum biochemical parameter of meat ducks is shown in Table 3. Compared with the 120 mg/kg MnSO_4_ group, serum TG concentration was significantly decreased in the 120 mg/kg MnHMet group (*P* < 0.05). Meanwhile, compared with 0 mg/kg MnHMet group, dietary supplementation with 60 and 120 mg/kg MnHMet had significantly higher serum HDL-C concentration (*P* < 0.05). In addition, as the levels of MnHMet increases, serum HDL-C exhibited a quadratic change (*P* < 0.05). However, there were no notable differences in the serum levels of NEFA, TC, and LDL-C among the different treatments (*P* > 0.05).

**Table 3:** Effects of hydroxy-methionine manganese on serum biochemistry of meat ducks.

**Items**	**Hydroxy-methionine manganese, mg/kg**						**MnSO_4_, mg/kg**	**SEM**	***P*** **-value**		
	**0**	**30**	**60**	**90**	**120**	**150**	**120**		**ANOVA**	**Linear**	**Quadratic**
NEFA	1.27	1.15	1.12	1.05	1.11	1.35	1.05	0.041	0.306	0.806	0.306
TG	0.70	0.63	0.59	0.55	0.53^B^	0.55	0.72^A^	0.018	0.056	0.004	0.134
TC	3.72	4.01	4.02	3.22	3.61	3.24	3.87	0.134	0.352	0.124	0.552
HDL-C	1.15^b^	1.33^ab^	1.65^a^	1.54^ab^	1.67^a^	1.18^b^	1.46	0.067	0.044	0.332	0.005
LDL-C	0.73	0.79	0.95	0.58	0.68	0.67	0.63	0.036	0.051	0.139	0.400

NEFA, Non-esterified fatty acid; TG, Triglyceride; TC, Total cholesterol; HDL-C, High-density lipoprotein cholesterol; LDL-C, Low-density lipoprotein cholesterol.

^a, b^Mean values (*n* = *6) in a row without a common superscript are significantly different in variance analysis (P* < *0.05)*.

^A, B^Mean values (*n* = 6) in a row without a common superscript are significantly different in T-test analysis (120 mg/kg as MnSO_4_ vs. 120 mg/kg as MnHMet) (*P* < 0.05).

### Meat quality

The effect of MnHMet on meat quality of meat ducks is shown in Table 4. Compared with 0 mg/kg group, dietary supplementation with 30, 90, 120, and 150 mg/kg MnHMet had significantly lower the drip loss of duck breast meat, and the drip loss was lowest in 90 mg/kg MnHMet group (*P* < 0.05). The drip loss of duck breast meat decreased linearly with increasing MnHMet levels (*P* < 0.05). Furthermore, the shear force of duck breast meat was lowest in the supplementation with 90 and 120 mg/kg MnHMet group than that in the CON group (*P* < 0.05), and the shear force decreased linearly and quadratically with increasing MnHMet levels (*P* < 0.05). Additionally, the IMF of duck breast meat in MnHMet groups was significantly higher than that in CON group (*P* < 0.05), and the IMF of duck breast meat decreased quadratically with increasing MnHMet levels (*P* < 0.05). However, no significant differences were observed in levels of pH_45min_, pH_24h_, L^*^, a^*^, and b^*^ among the groups (*P* > 0.05).

**Table 4:** Effects of hydroxy-methionine manganese on meat quality of meat ducks.

**Items**	**Hydroxy-methionine manganese, mg/kg**						**MnSO_4_, mg/kg**	**SEM**	***P*** **-value**		
	**0**	**30**	**60**	**90**	**120**	**150**	**120**		**ANOVA**	**Linear**	**Quadratic**
pH _45min_	5.91	5.94	5.93	5.45	6.08	5.97	5.95	0.221	0.064	0.193	0.065
pH _24h_	5.27	5.29	6.00	6.06	6.04	5.97	5.97	0.176	0.584	0.121	0.408
*L* ^*^_45min_	54.56	56.27	55.60	52.37	52.28	52.91	52.4	0.640	0.320	0.076	0.827
*a* ^*^_45min_	13.46	13.52	12.96	14.44	14.68	15.52	15.55	0.285	0.110	0.005	0.252
*b* ^*^_45min_	7.97	8.81	8.49	8.58	8.59	8.70	9.18	0.155	0.715	0.354	0.516
Drip loss, %	4.14^a^	2.86^bc^	3.70^ab^	2.27^c^	2.80^bc^	2.65^bc^	3.03	0.002	0.014	0.008	0.225
Shear force, kg	6.51^a^	5.82^ab^	5.49^ab^	4.96^bc^	4.46^c^	5.99^ab^	5.01	0.189	0.016	0.044	0.008
IMF, %	3.76^b^	4.65^a^	4.78^a^	4.90^a^	4.54^a^	4.45^a^	4.68	0.101	0.009	0.075	0.001

L^*^, Lightness; a^*^, Redness; b^*^, Yellowness; IMF, Intramuscular fat.

^a, b, c^Mean values (*n* = 6) in a row without a common superscript are significantly different in variance analysis (*P* < 0.05).

### Carcass quality

As indicated in Table 5, compared with the 0 mg/kg MnHMet group, dietary supplementation with 90 and 120 mg/kg MnHMet significantly reduced the abdominal fat weight and abdominal fat percentage of duck breast meat, and the lowest values for abdominal fat weight and percentage were observed in the 120 mg/kg MnHMet group (*P* < 0.05). Additionally, the abdominal fat weight and abdominal fat percentage in meat duck decreased linearly and quadratically with increasing MnHMet levels (*P* < 0.05). However, dietary supplementation with MnHMet did not show a significant effect on other indicators of breastmuscle (*P* > 0.05).

**Table 5:** Effects of hydroxy-methionine manganese on carcass quality of meat ducks.

**Items**	**Hydroxy-methionine manganese, mg/kg**						**MnSO_4_, mg/kg**	**SEM**	***P*** **-value**		
	**0**	**30**	**60**	**90**	**120**	**150**	**120**		**ANOVA**	**Linear**	**Quadratic**
Carcass weight, kg	2.04	2.00	1.98	1.97	2.04	2.03	2.00	0.012	0.377	0.855	0.052
Half carcass weight, kg	1.91	1.84	1.83	1.83	1.90	1.86	1.86	0.012	0.230	0.771	0.076
Eviscerated weight, kg	1.78	1.72	1.70	1.67	1.74	1.70	1.69	0.011	0.150	0.139	0.067
Breast muscle weight, g	166.17	157.50	159.88	157.21	157.95	175.65	171.19	2.890	0.329	0.449	0.069
Thigh muscle weight, g	229.71	232.88	228.06	224.26	219.78	214.39	231.77	3.641	0.730	0.125	0.623
Abdominal fat weigh, g	20.03^a^	17.24^ab^	14.23^ab^	12.29^b^	11.65^b^	14.33^ab^	13.03	0.865	0.033	0.005	0.043
Dressing percentage, %	89.00	89.61	90.90	87.95	87.03	87.23	86.63	0.005	0.230	0.067	0.339
Half carcass weight rate, %	80.76	82.47	83.64	82.74	80.00	79.39	80.63	0.006	0.307	0.241	0.060
Eviscerated carcass weight rate, %	76.97	76.87	76.69	74.85	74.28	74.33	73.15	0.006	0.510	0.241	0.950
Breast muscle rate, %	9.25	9.21	9.40	9.11	9.07	10.31	10.10	1.548	0.130	0.155	0.120
Thigh muscle rate, %	12.53	13.58	13.42	13.51	12.65	12.66	13.74	2.309	0.595	0.675	0.134
Abdominal fat percentage, %	1.13^a^	1.00^ab^	0.83^ab^	0.74^b^	0.67^b^	0.89^ab^	0.76	0.001	0.026	0.010	0.025

^a, b^Mean values (*n* = 6) in a row without a common superscript are significantly different in variance analysis (*P* < 0.05).

### Fatty acid composition in liver

In Table 6, the effect of MnHMet on the fatty acid composition in the liver of meat ducks is demonstrated. Liver C23:0 showed a quadratic decrease as the levels of MnHMet increased. Specifically, compared with other groups, adding 60 and 120 mg/kg MnHMet to the diet significantly reduced liver C23:0 (*P* < 0.05). However, no significant differences were detected in other liver fatty acid composition among each of the groups (*P* > 0.05).

**Table 6:** Effects of hydroxy-methionine manganese on fatty acid composition in liver of meat ducks.

**Items, %**	**Hydroxy-methionine manganese, mg/kg**						**MnSO_4_, mg/kg**	**SEM**	***P*** **-value**		
	**0**	**30**	**60**	**90**	**120**	**150**	**120**		**ANOVA**	**Linear**	**Quadratic**
C16:0	28.53	24.80	24.53	24.47	25.90	28.25	26.42	0.026	0.086	0.857	0.005
C16:1	1.05	1.03	1.38	1.18	1.28	1.25	1.25	0.003	0.621	0.297	0.449
C17:0	0.35	0.33	0.50	0.35	0.35	0.28	0.28	0.002	0.615	0.551	0.257
C18:0	18.83	18.05	18.00	19.47	16.18	15.95	17.08	0.021	0.295	0.604	0.607
C18:1 n9t	0.20	0.25	0.25	0.20	0.23	0.23	0.23	0.001	0.431	0.996	0.467
C18:1 n9c	27.23	33.93	25.27	31.77	31.98	30.47	31.40	0.049	0.223	0.433	0.815
C18:2n6t	1.50	1.93	9.65	3.00	0.80	0.23	0.83	0.063	0.313	0.529	0.128
C18:2n6c	10.8	9.20	9.83	10.68	10.83	12.47	10.02	0.027	0.108	0.037	0.051
C18:3n6	0.28	0.35	0.30	0.30	0.45	0.43	0.35	0.002	0.051	0.010	0.425
C18:3n3	0.33	0.30	0.58	0.38	0.45	0.45	0.40	0.002	0.288	0.244	0.407
C20:2	0.33	0.30	0.43	0.33	0.37	0.45	0.63	0.001	0.425	0.162	0.803
C20:0	0.10	0.13	0.08	0.10	0.08	0.05	0.10	0.001	0.232	0.049	0.451
C20:3n6	1.25	1.03	1.13	1.30	1.00	1.10	1.02	0.002	0.354	0.486	0.994
C20:5n3	0.25	0.20	0.25	0.20	0.18	0.20	0.15	0.001	0.446	0.160	0.792
C22:0	0.83	0.60	0.68	0.70	0.60	0.55	0.70	0.002	0.561	0.158	0.864
C22:6n3	0.20	0.13	0.20	0.17	0.18	0.13	0.30	0.001	0.200	0.186	0.522
C23:0	7.67^a^	7.03^a^	5.13^b^	7.03^a^	4.93^b^	6.93^a^	5.77	0.013	0.019	0.107	0.031
C24:0	0.20	0.15	0.10	0.15	0.13	0.10	0.15	0.001	0.229	0.055	0.433
SFA	55.53	50.73	52.25	51.83	50.23	51.05	51.05	0.122	0.882	0.386	0.608
MUFA	1.10	2.43	1.37	1.23	1.33	1.25	1.43	0.011	0.599	0.574	0.648
PUFA	42.78	46.08	44.43	46.15	47.68	47.08	45.50	0.134	0.933	0.363	0.839

Saturated fatty acid (SFA) = C16:0 + C17:0 + C18:0 + C20:0 + C22:0+ C23:0 + C24:0. Monounsaturated fatty acid (MUFA) = C16:1 + C18:1n9t + C18: 1 n9c. Polyunsaturated fatty acid (PUFA) = C18:2n6t + C18:2n6c + C18:3n3 + C18:3n6 + C20:2 + C20:3n6 + C20:5n3 + C22:6n3.

^a, b^Mean values (*n* = 6) in a row without a common superscript are significantly different in variance analysis (*P* < 0.05).

### Antioxidant capacity in breast muscle and liver

**Figure 1:**
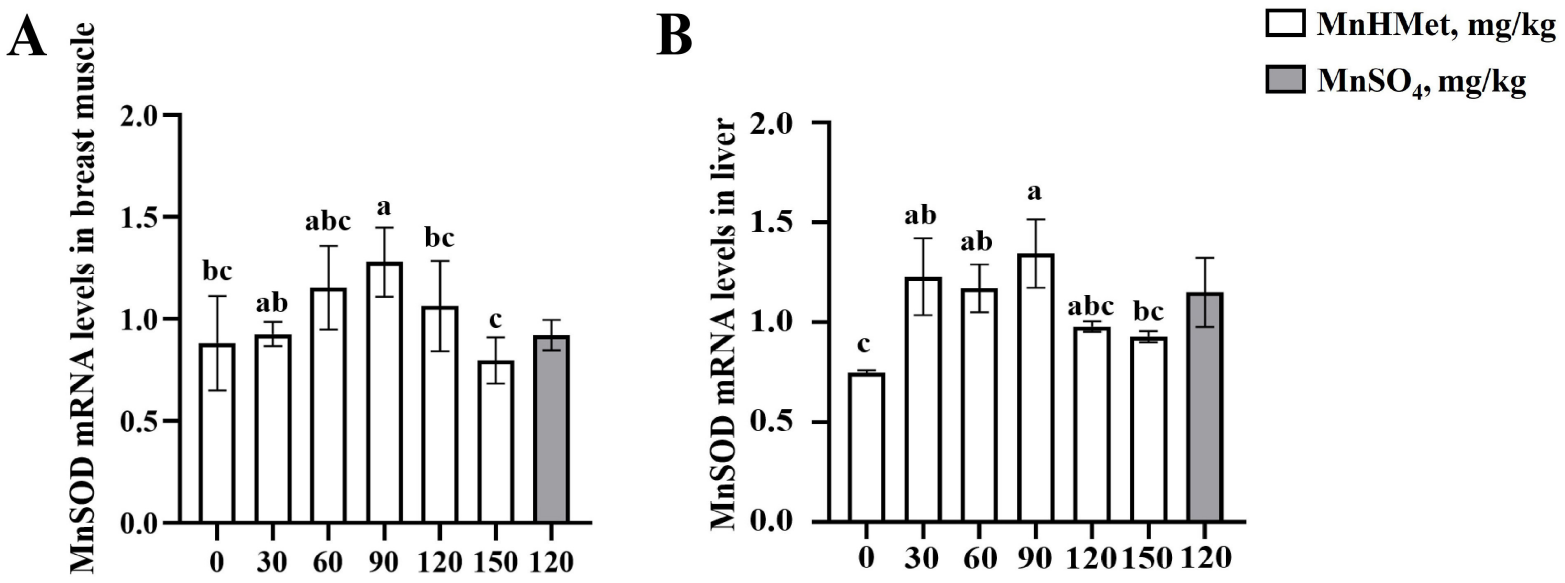
Effects of hydroxy-methionine manganese on the expression of manganese superoxide dismutase in breast muscle and liver of meat ducks. **(A, B)** Manganese superoxide dismutase (MnSOD). Bars with different letters indicate *P* < 0.05. Values are presented as mean and standard error of mean (*n* = 6).

The breast muscle antioxidant indexes are depicted in Table 7 and Figure 1A. Dietary MnHMet supplementation significantly increased the levels of T-AOC in breast muscle with quadratic change (*P* < 0.05). Dietary supplementation with 60 mg/kg MnHMet significantly increased the levels of T-AOC in breast muscle compared with 0 and 120 mg/kg MnHMet groups (*P* < 0.05). Besides, compared with 120 mg/kg MnSO_4_ group, the breast muscle level of CAT was significantly higher in 120 mg/kg MnHMet group (*P* < 0.05). There were no significant differences in breast muscle levels of MnSOD and MDA among the groups (*P* > 0.05). In addition, Figure 1A showed that dietary supplementation with 90 mg/kg MnHMet significantly increased *MnSOD* mRNA expression compared to the 0, 120, and 150 mg/kg MnHMet group (*P* < 0.05).

**Table 7:** Effects of hydroxy-methionine manganese on antioxidant capacity in breast muscle of meat ducks.

**Items**	**Hydroxy-methionine manganese, mg/kg)**						**MnSO_4_, mg/kg**	**SEM**	***P*** **-value**		
	**0**	**30**	**60**	**90**	**120**	**150**	**120**		**ANOVA**	**Linear**	**Quadratic**
MnSOD, U/mg protein	51.84	55.85	57.71	52.25	47.95	55.58	53.10	1.531	0.515	0.742	0.856
T-AOC, U/mg protein	0.12^b^	0.21^ab^	0.31^a^	0.21^ab^	0.16^b^	0.21^ab^	0.21	0.016	0.014	0.600	0.015
MDA, nmol/mg protein	1.06	0.82	1.01	1.00	1.40	1.05	1.57	0.068	0.390	0.249	0.841
CAT, U/mg protein	256.94	260.84	258.73	296.81	257.7^A^	189.04	165.50^B^	8.957	0.130	0.134	0.024

MnSOD, Manganese superoxide dismutase; T-AOC, Total antioxidant capacity; MDA, Malondialdehyde; CAT, Catalase.

^a, b^Mean values (*n* = 6) in a row without a common superscript are significantly different in variance analysis (*P* < 0.05).

^A, B^Mean values (*n* = 6) in a row without a common superscript are significantly different in T-test analysis (120 mg/kg as MnSO_4_ vs. 120 mg/kg as MnHMet) (*P* < 0.05).

The hepatic antioxidant related indexes are depicted in Table 8 and Figure 1B. Compared to the 0 mg/kg MnHMet group, dietary supplementation with 90, 120, and 150 mg/kg of MnHMet significantly elevated MnSOD levels in the liver (*P* < 0.05). Supplementation with 90 and 120 mg/kg of MnHMet enhanced T-AOC levels in liver than that in 0 mg/kg MnHMet group (*P* < 0.05). Meanwhile, both MnSOD and T-AOC levels demonstrated a linear increase with dietary MnHMet increasing in the liver of meat ducks (*P* < 0.05). Moreover, dietary MnHMet supplementation significantly reduced the liver MDA level with quadratic change (*P* < 0.05). The addition of 60 mg/kg MnHMet in the meat duck diet significantly decreased liver MDA levels (*P* < 0.05). Additionally, compared with the MnSO_4_ group, the liver MDA level was significantly lower in 120 mg/kg MnHMet group (*P* < 0.05). The liver CAT level did not show any significant differences among the groups (*P* > 0.05). In addition, in Figure 1B, dietary supplementation with 30, 60, and 90 mg/kg MnHMet significantly up-regulated the mRNA expression of *MnSOD* compared to the 0 mg/kg MnHMet group (*P* < 0.05).

**Table 8:** Effects of hydroxy-methionine manganese on antioxidant capacity in liver of meat ducks.

**Items**	**Hydroxy-methionine manganese, mg/kg**						**MnSO_4_, mg/kg**	**SEM**	***P*** **-value**		
	**0**	**30**	**60**	**90**	**120**	**150**	**120**		**ANOVA**	**Linear**	**Quadratic**
MnSOD, U/mg protein	43.59^b^	51.04^ab^	51.27^ab^	62.48^a^	56.82^a^	57.69^a^	54.96	1.833	0.037	0.060	0.128
T-AOC, U/mg protein	1.28^b^	1.20^b^	1.46^ab^	1.58^a^	1.61^a^	1.42^ab^	1.60	0.041	0.010	0.007	0.063
MDA, nmol/mg protein	3.23^ab^	2.95^bc^	2.84^c^	2.98^bc^	3.32^aB^	3.17^ab^	3.89^A^	0.049	0.017	0.270	0.013
CAT, U/mg protein	585.34	596.90	612.07	613.78	629.27	621.57	636.38	5.341	0.182	0.012	0.419

MnSOD, Manganese superoxide dismutase; T-AOC, Total antioxidant capacity; MDA, Malondialdehyde; CAT, Catalase.

^a, b, c^Mean values (*n* = 6) in a row without a common superscript are significantly different in variance analysis (*P* < 0.05).

^A, B^Mean values (*n* = 6) in a row without a common superscript are significantly different in T-test analysis (120 mg/kg as MnSO_4_ vs. 120 mg/kg as MnHMet) (*P* < 0.05).

### Abdominal adipocyte

**Figure 2:**
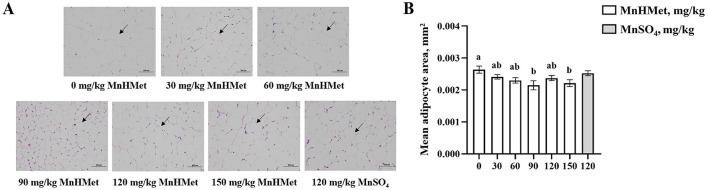
Effects of hydroxy-methionine manganese on abdominal adipocytes in meat ducks. **(A)** Representative images (×100) were stained with hematoxylin and eosin; **(B)** The average area of adipocyte. Bars with different letters indicate *P* < 0.05. Values are presented as mean and standard error of mean (*n* = 6).

The effects of MnHMet on abdominal adipocytes are shown in Figure 2, compared with the 0 mg/kg MnHMet group, dietary supplementation with MnHMet had significantly lower the abdominal adipocyte area of meat ducks, and the abdominal adipocyte area was lowest in 90 mg/kg MnHMet group (*P* < 0.05). Nevertheless, no differences in abdominal adipocytes were observed between the 120 mg/kg MnHMet group and 120 mg/kg MnSO_4_ group (*P* > 0.05).

### mRNA expression of lipid metabolism related gene

**Figure 3:**
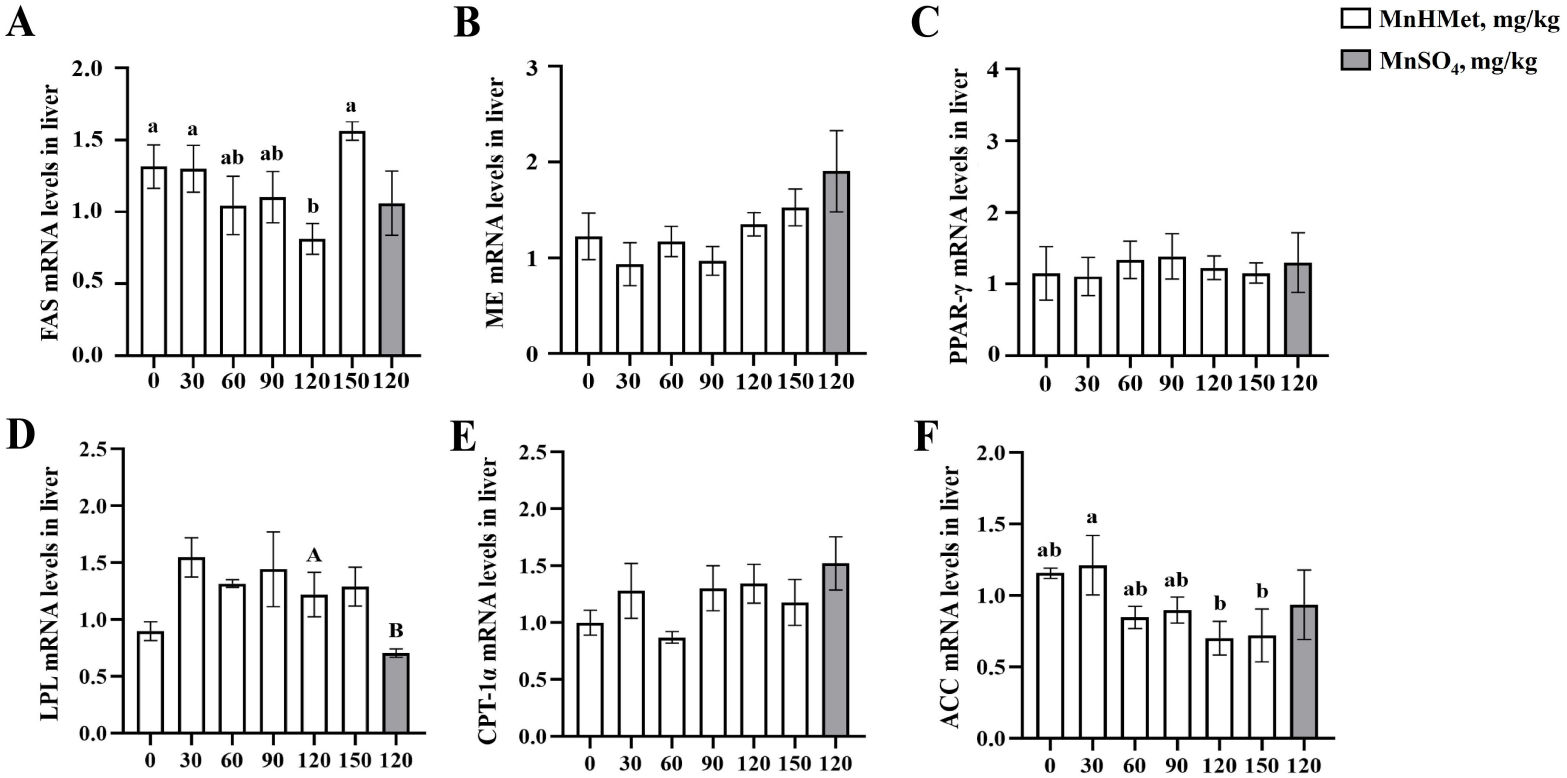
Effects of hydroxy-methionine manganese on gene expressions of lipid metabolism in liver of meat ducks. **(A)** Fatty acid synthase (FAS); **(B)** Malic enzyme (ME); **(C)** Peroxisome proliferator activated receptor-γ (PPAR-γ); **(D)** Lipoprotein lipase (LPL); **(E)** Carnitine palmitoyltransterase-1α (CPT-1α); **(F)** Acetyl-CoA carboxylase (ACC). Bars with different letters indicate *P* < 0.05. Values are presented as mean and standard error of mean (*n* = 6).

Gene expression related to lipid metabolism in liver is displayed in Figure 3. Dietary supplementation with 120 mg/kg MnHMet significantly decreased fatty acid synthase (*FAS*) mRNA expression compared to the 0 mg/kg MnHMet group (*P* < 0.05). Moreover, 120 and 150 mg/kg MnHMet supplementation led to a down-regulation of acetyl-CoA carboxylase (*ACC*) mRNA expression (*P* < 0.05). On the other hand, hepatic lipoprotein lipase (*LPL*) mRNA expression was notably increased in the 120 mg/kg MnHMet group when compared to the group supplemented with 120 mg/kg MnSO_4_ (*P* < 0.05). Notably, there were no significant differences observed in the mRNA expressions of peroxisome proliferator activated receptor-γ (*PPAR-*γ), malic enzyme (*ME*), and carnitine palmitoyltransferase-1α (*CPT-1*α) among the groups (*P* > 0.05).

**Figure 4:**
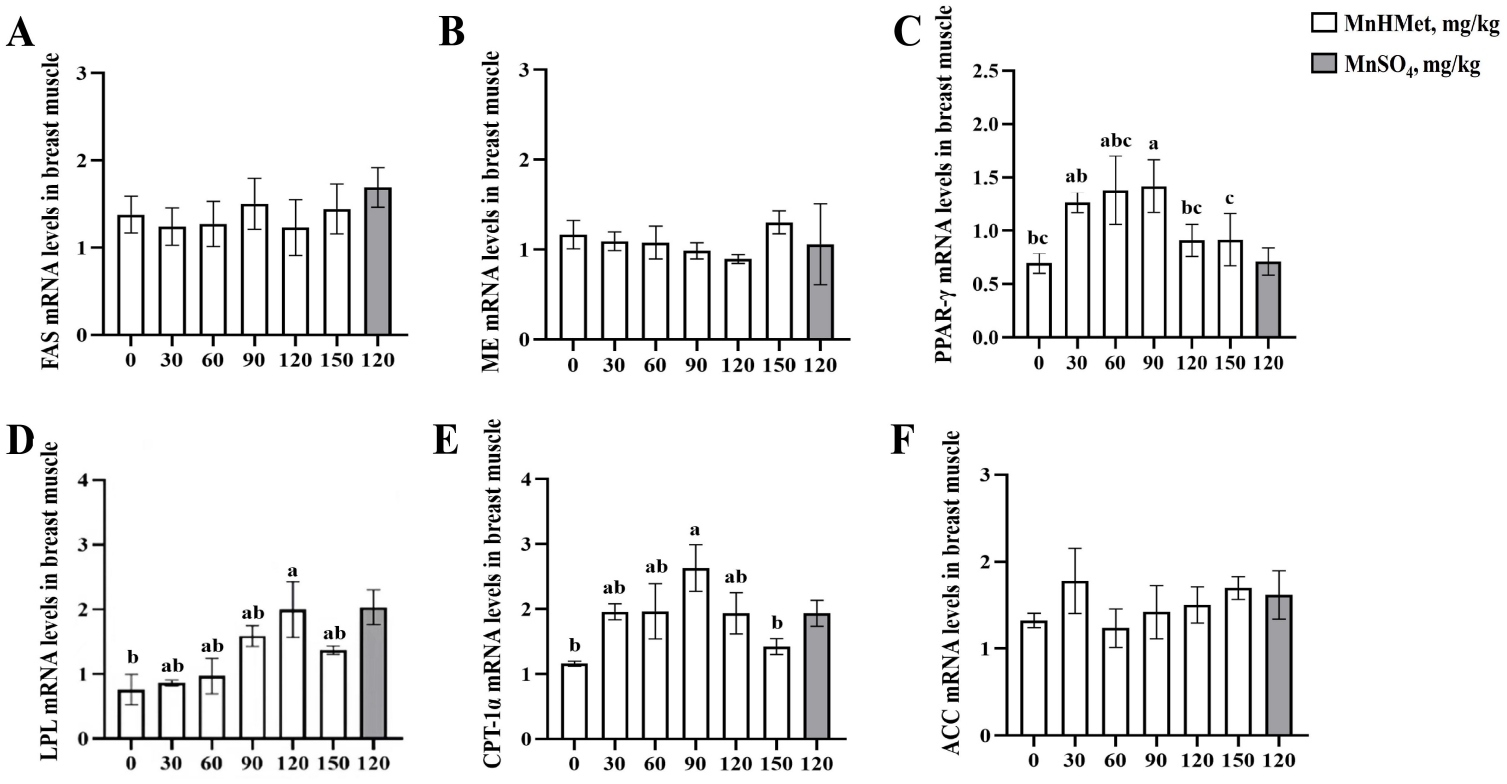
Effects of hydroxy-methionine manganese on gene expressions of lipid metabolism in breast muscle of meat ducks. **(A)** Fatty acid synthase (FAS); **(B)** Malic enzyme (ME); **(C)** Peroxisome proliferator activated receptor-γ (PPAR-γ); **(D)** Lipoprotein lipase (LPL); **(E)** Carnitine palmitoyltransterase-1α (CPT-1α); **(F)** Acetyl-CoA carboxylase (ACC). Bars with different letters indicate *P* < 0.05. Values are presented as mean and standard error of mean (*n* = 6).

Gene expression related to lipid metabolism in duck breast is displayed in Figure 4. In the duck breast, supplementation with 90 mg/kg MnHMet significantly increased the expression of *PPAR-*γ and *CPT-1*α mRNA when compared to the 0 mg/kg MnHMet group (*P* < 0.05). Furthermore, the 120 mg/kg MnHMet group exhibited significantly higher *LPL* mRNA expression in the duck breast compared to the 0 mg/kg MnHMet group (*P* < 0.05). Notably, no significant differences in the mRNA expressions of *FAS, ME*, and *ACC* among the groups were observed (*P* > 0.05).

### Estimation of the dietary MnHMet requirements

The optimal MnHMet supplementation levels based on different effect indexes are presented in Table 9. Under the conditions of this experiment, quadratic regression analysis and the establishment of univariate quadratic equations indicated that the optimal MnHMet supplementation in the basal diet for meat ducks was 107.5 mg/kg when using abdominal fat percentage as the effect index, and 117.5 mg/kg when using IMF as the effect index.

**Table 9:** The optimal dietary hydroxy-methionine manganese supplementation based on different indices for ducks.

**Items**	**Regression equation^a^**	***R* ^2^**	***P*-value**	**Optimal dietary Mn-Met level (mg/kg diet)**
Abdominal fat percentage	y = 4E-0.5x^2^−0.0086x+1.1675	0.903	<0.05	107.5
Intramuscular fat	y = −0.0001x^2^+0.0235x+3.8101	0.935	<0.05	117.5

a y is the dependent variable and x are the dietary Mn-Met supplemental levels (mg/kg).

## Discussion

Manganese is an essential micronutrient for the growth and development of poultry (30). Organic manganese chelates, compared to manganese sulfate, have demonstrated higher bioavailability and can more effectively enhance growth performance (31). Growth performance is the gold standard for evaluating the effect of feed additives (32). In this study, we found that with the increase of MnHMet levels, the F/G from 15 to 35 days showed linear and quadratic decreases, among which the F/G of meat ducks fed with 120 mg/kg MnHMet significantly decreased. A study in chickens showed that dietary organic manganese supplementation benefited the growth performance (20), and another study in chickens showed that dietary organic Mn supplementation reduced the F:G in broilers (33). These findings suggest that organic manganese may have a beneficial effect on improving animal production performance. However, dietary supplementation with MnHMet did not observe significant effects on other growth performance indicators. In contrast, other studies in broilers did not observe significant changes in growth performance when fed a corn-soybean meal diet supplemented with Mn (34). Similarly, a study on supplementing inorganic manganese in meat duck feed found no significant effect of MnSO_4_ addition on the growth performance of meat ducks (15). Therefore, based on our research, we believe that the abnormal decrease in F/G from days 15 to 35 in the 120 mg/kg MnHMet group may be a special case, and not necessarily reflective of a general trend in MnHMet supplementation.

Drip loss, shear force, and intramuscular fat content are key indicators to assess the meat quality of livestock (35). Higher drip loss and shear force typically indicate lower water holding capacity and tenderness, while intramuscular fat content is a critical factor for assessing nutritional value and juiciness (36). Previous research has shown that adding MnSO4 to duck diets can significantly increase IMF content and decrease drip loss and shear force in breast muscle (15). Li et al. (37) also proved that dietary organic Mn supplementation reduced the shear force, drip loss and pressure loss in breast muscle of chickens. In our current study, dietary supplementation with MnHMet similarly resulted in a significant increase in IMF content, along with reduced drip loss in the 90 mg/kg MnHMet group and decreased shear force in the 120 mg/kg MnHMet group of breast muscle, suggesting that MnHMet can effectively enhance meat quality. Meanwhile, the improvements in meat quality were more pronounced with higher levels of MnHMet supplementation. In conclusion, dietary MnHMet supplementation could improve the water holding capacity, nutritional value, and tenderness of duck breast, and meat quality was more significantly improved with the increase of MnHMet supplemental level.

Antioxidative or oxidative stress are important indicators to measure the condition of the animal complex (38). Oxidative stress intensifies and contributes to the ER unfolded protein response, which in turn affects protein synthesis (32). Oxidative capacity is a critical measure of muscle quality in meat, as lipid and protein oxidation can degrade its flavor, color, and nutritional value (11). Manganese plays a crucial role in antioxidant activity, reducing oxidative stress-induced lipid peroxidation (15, 39, 40). Superoxide dismutase (SOD) is widely regarded as the first line of defense against oxidative damage, which mainly exists in two forms: CuZn-SOD and Mn-SOD (23). As a cofactor of MnSOD, Mn could protect mitochondria from oxidative damage by catalyzing the conversion of superoxide anions to hydrogen peroxide (41). A hybrid grouper study indicated that supplementation with MnMet could enhance the activities of T-SOD, Mn-SOD, and GSH-PX in the liver of grouper as dietary Mn levels increased (42). Besides, several studies have demonstrated that supplementing diets with manganese increases the activities of MnSOD, CAT, GSH-Px, and T-AOC in the serum or tissues of broiler chickens (13, 43, 44). The antioxidant defense system includes various components, such as T-AOC and CAT, and elevated levels of these antioxidant factors reflect an improved ability of the body to scavenge free radicals (45, 46). Our study found that dietary supplementation with 90 mg/kg MnHMet significantly increased levels of T-AOC and MnSOD mRNA expression in the breast muscle and liver, along with elevated MnSOD levels in the liver. Meanwhile, our study indicates that adding high levels of manganese to the diet can lead to a decrease in antioxidant related indicators in the liver or muscle, consistent with the results of Liu et al. (42). MDA is a well-known marker for lipid peroxidation, indicating oxidative stress (47, 48). Hepatic MDA level in 90 mg/kg MnHMet group was also lower than that in other treatments in the present study. Meanwhile, compared to MnSO4 group, supplementation with 120 mg/kg MnHMet resulted in higher CAT levels in the breast muscle and lower MDA levels in the liver. The increase of CAT and SOD was associated with Nrf2, and with decreased MDA. Therefore, MnHMet has a better effect on improving the antioxidant capacity of meat ducks than inorganic manganese. Overall, our findings suggest that MnHMet supplementation enhances the antioxidant capacity in the liver and breast muscle of meat ducks, offering potential advantages over traditional MnSO4 supplementation.

Lipid metabolism involves the synthesis, degradation, and transportation processes of fatty substances in the body, which are crucial for maintaining health and energy balance (49). Research indicates that Mn influences various aspects of lipid metabolism (50). In the current study, we found that adding 90 or 120 mg/kg MnHMet to the diet significantly reduced the abdominal fat weight and abdominal fat of meat ducks. Furthermore, HE staining of abdominal fat revealed that the surface area of adipocytes was smaller in ducks fed MnHMet. Serum biochemical indicators, such as TG, TC, LDL-C, and HDL-C, are vital for assessing lipid metabolism and overall health (51). In this study, we found that supplementation with 60 mg/kg or 120 mg/kg MnHMet in the diet of meat ducks could increase serum HDL-C levels. HDL-C can promote the TG from peripheral tissues to the liver for catabolism (52). Additionally, we observed significantly lower serum TG levels in meat ducks supplemented with organic Mn compared to supplemented with inorganic Mn, which was consistent with the results by Zhang et al. (53). Fatty acids are important components of lipid metabolism and closely related to it. In poultry, the liver is an essential organ for lipid metabolism in poultry and an integral part of de novo fatty acid synthesis, accounting for about 95% (54, 55). In our study, we observed that dietary supplementation with 60 or 120 mg/kg MnHMet decreased the levels of tricosanoic acid (C23:0) in liver of meat ducks. Tricosanoic acid (C23:0) is a saturated fatty acid that can lead to hepatic lipid metabolism disorder (56). Taken together, these results indicate that dietary MnHMet is beneficial for reducing abdominal fat deposition and regulating lipid metabolism and liver fatty acids in meat ducks.

To identify possible mechanisms of dietary MnHMet in regulating lipid metabolism, we measured gene expression related to lipid metabolism in both the liver and breast muscles (15). In the current study, dietary supplementation with 90 mg/kg or 120 mg/kg MnHMet could increase mRNA expressions of breast muscle *PPAR-*γ and *LPL*. Meanwhile, compared to the adding MnSO_4_ group, the expression level of *LPL* mRNA in the liver of meat ducks in the 120mg/kg MnHMet group was significantly higher than that in the MnSO_4_ group. Our consistent results with Yang et al. showed that adding manganese to diets increased the mRNA expressions of PPAR-γ and LPL activity in breast muscle of meat ducks (15). PPAR-γ, a nuclear hormone receptor, promotes adipocyte differentiation and lipid metabolism by increasing LPL expression, which catalyzes the breakdown of triglycerides for tissue oxidation and energy storage (57, 58). Therefore, our results indicate that adding MnHMet to the diet of meat ducks can affect the utilization of fatty acids by upregulating the expression of muscle *PPAR*γ and *LPL*, thereby promoting intramuscular fat deposition. Interestingly, adding 90 mg/kg MnHMet to the diet of meat ducks has also been found to lead to an increase in the mRNA expression of muscle CPT-1 α. Research has shown that CPT-1α can facilitate fatty acid oxidation by enabling their transport across mitochondrial membranes for β-oxidation (59, 60). When the expression of PPARγ and LPL in muscles increases, leading to an increase in fatty acid uptake, the expression of CPT-1 α also increases, which may be an adaptive response of muscle tissue. Muscle cells may process more fatty acids by enhancing their oxidative capacity, thereby maintaining metabolic balance and preventing excessive fat deposition (61). In addition, ACC and FAS are the key lipogenic enzymes, our study also found that the dietary supplementation with 120 mg/kg MnHMet could decrease the mRNA expressions of *FAS* and *ACC* in liver of meat ducks. FAS, which catalyzes the final step in the lipogenic pathway, is a critical determinant of a tissue's capacity to synthesize fatty acids (62, 63). Previous research by Lu et al. found that manganese decreased FAS activity in primary broiler hepatocytes (64). Our results demonstrated that the addition of MnHMet decreased the expression of lipid synthesis related genes *ACC* and *FAS* in the liver of meat ducks, which could reduce abdominal fat deposition in meat ducks. In summary, these findings indicate that dietary MnHMet appears to be more effective than MnSO4 in regulating lipid metabolism by influencing the expression of genes involved in fatty acid synthesis, transport, and oxidation in both the breast muscle and liver, contributing to improved fat accumulation and metabolic balance.

## Conclusion

In summary, our research indicates that adding MnHMet to the diet of meat ducks enhances growth performance, meat quality, antioxidant capacity, and fatty acid composition, while reducing abdominal fat deposition by regulating the expression of genes involved in muscle and liver lipid metabolism, thereby enhancing the lipid metabolism capacity of meat ducks. Dietary supplementation with 107.5 or 117.5 mg/kg MnHMet in the diet of meat ducks is recommended to decrease abdominal fat percentage and improve intramuscular fat content, respectively. Furthermore, our research confirms that organic Mn has a more significant impact on meat duck production compared to inorganic Mn.

## Data Availability

The original contributions presented in the study are included in the article/Supplementary material, further inquiries can be directed to the corresponding author.
