# Supplementary material for: Dietary supplementation with hydroxy-methionine manganese improves meat quality, antioxidant capacity, and lipid metabolism in Cherry Valley ducks (Anas platyrhynchos domesticus)
Source: Front Vet Sci. 2025 Apr 4;12:1481793. doi: 10.3389/fvets.2025.1481793 (PMC12007453; doi:10.3389/fvets.2025.1481793)
Supplement: Supplementary file 1 [file Data_Sheet_1.pdf]

# *Supplementary Material*

## Supplementary Tables

**Table S1. Analyzed manganese concentration of diets (air-dry basis)**

| Items | Dietary manganese level, mg/kg | Analyzed manganese, mg/kg | |
|---|---|---|---|
| | | 1-14 d | 15-35 d |
| Hydroxy-methionine manganese | 0 | 28.45 | 30.12 |
| | 30 | 57.41 | 58.72 |
| | 60 | 91.09 | 93.65 |
| | 90 | 118.59 | 119.92 |
| | 120 | 147.43 | 150.20 |
| | 150 | 182.17 | 183.62 |
| $MnSO_4$ | 120 | 146.87 | 148.21 |

**Table S2. The primers for quantitative real-time PCR**

| Gene name [a] | GenBank | Primer sequences, 5′-3′ [b] | Size, bp |
|---|---|---|---|
| *MnSOD* | XM_027454289.1 | F: GCTGCAAAAGGTGATGTTACA<br>R: TTGCAAAGGAACAAAGTCACG | 179 |
| *LPL* | FJ185781.1 | F: AGTACGCTGAGCCCTTACG<br>R: AGCAATCAGACGCAGAGCTT | 191 |
| *ACC* | XM_027472449.1 | F: TAAGATGCACCTCTACCTGGGA<br>R: TTAAATGCCACCTCCAGCTCAT | 189 |
| *FAS* | XM_027455970.1 | F: GCCTGTGGTTTTATGCCACT<br>R: ACCTCCTGAGCCAGAGTGAA | 184 |
| *CPT-1α* | XM_027457808.1 | F: ATCAACTGTTCCGCTCTGCT<br>R: CAACGATGTGCTTGCTGTCT | 113 |
| *ME* | KF185113.1 | F: GACCCTCACCTCAACAAGGG<br>R: CGAGAAAGCAAGGTGGCAAT | 228 |
| *PPARγ* | NM_001310398.1 | F: GCCCAAGTTTGAGTTCGCTG<br>R: AACAGCTGTGACGACTCTGG | 201 |
| *β-actin* | NM_001310421.1 | F: CCAGCCATCTTTCTTGGGTA<br>R: GTGTTGGCGTACAGGTCCTT | 105 |

[a] MnSOD = manganese superoxide dismutase; LPL = lipoprotein lipase; ACC = acetyl-CoA carboxylase; FAS = fatty acid synthase; CPT-1α = carnitine palmitoyltransferase-1α; ME = malic enzyme; PPAR-γ = peroxisome proliferator activated receptor-γ.

[b] F: forward primer; R: reverse primer
